# Automatic Detection of Extracochlear Electrodes in Cochlear Implants Using Electric Field Imaging: Bench and Clinical Validation

**DOI:** 10.3390/audiolres16040119

**Published:** 2026-08-14

**Authors:** Mehrangiz Ashiri, Tony Spahr, Chen Chen, Azret Botash, Ashish Mehta, Patrick Boyle, Manohar Bance, Daniele Borsetto, Susan T. Eitutis, Jordan J. Varghese, Craig A. Buchman, René H. Gifford, Andrea J. DeFreese, Matthew Miller, Syed F. Ahsan, Christopher Danner, Kyle P. Allen, Loren Bartels, Kanthaiah Koka

**Affiliations:** 1 Research and Technology, Advanced Bionics LLC, Valencia, CA 91355, USA; 2 Auditory Neuroscience Lab, Barrow Neurological Institute, Phoenix, AZ 85013, USA; 3 Department of Clinical Neurosciences, University of Cambridge, Cambridge CB2 0QQ, UK; 4 Department of Otolaryngology, Washington University, St. Louis, MO 63130, USA; 5 Department of Otolaryngology—Head and Neck Surgery, University of Utah, Salt Lake City, UT 84132, USA; 6 Hearts for Hearing, Oklahoma City, OK 73120, USA; 7 Department of Hearing and Speech Sciences, Vanderbilt University Medical Center, Nashville, TN 37232, USA; 8 Otolaryngology-Head and Neck Surgery, Sanford Health, Fargo, ND 58104, USA; 9 Head & Neck Surgery Department, Southern California Kaiser Permanente Medical Group, Anaheim, CA 92806, USA; 10 Tampa Bay Hearing & Balance Center, Tampa, FL 33606, USA

**Keywords:** cochlear implant (CI), extracochlear electrode (EE), electric field imaging (EFI), impedance

## Abstract

Objectives: Extracochlear electrodes (EEs), defined as electrode contacts located outside the cochlea, can degrade cochlear implant (CI) performance. We present and validate an Electric Field Imaging (EFI)-based algorithm that helps detect EEs and can be used intra- or post-operatively. Methods: An algorithm for detecting EEs from EFI data was developed. Validation and testing were performed using three datasets: (a) bench models with known EE conditions (saline and resistive load model), (b) clinical EFI recordings from CI recipients with imaging confirmation (CT or plain X-ray), and (c) EFI recordings without imaging. Primary outcomes included the ability to differentiate extracochlear conditions from fully inserted electrode arrays, as well as the concordance between the number of EE contacts identified by the algorithm and those determined from controlled bench configurations or available imaging data. Results: The algorithm reliably differentiated full insertion from EE conditions on bench models and clinical EFI recordings from CI recipients with imaging confirmation. In the imaging-confirmed clinical cohort (6 EE cases among 226 CI recipients), the algorithm achieved 100% sensitivity and specificity. In clinical cases without imaging, the algorithm flagged 3.94% as having extracochlear electrodes, within the range of prevalence reported in the literature. Furthermore, the lateral-wall electrode arrays were associated with a significantly higher extracochlear electrode occurrence compared to the pre-curved arrays. Conclusions: This EFI-based algorithm may provide a useful screening tool for EEs using routine clinical measurements, supporting intra-operative and post-operative detection while reducing reliance on imaging and thereby aiding clinical decision-making. The method can be integrated into existing clinical fitting software workflow and complements recent work on EFI-based tip fold-over detection. The observed sensitivity and specificity suggest that this approach may provide a valuable tool for identifying EEs in situations where imaging is unavailable or impractical.

## 1. Introduction

Optimal intracochlear placement of the electrode array is critical to maximize benefit from a cochlear implant (CI). Electrodes residing outside the cochlea, termed extracochlear electrodes (EEs), may result from incomplete insertion, post-operative electrode array migration (e.g., during closure of the skin, or with head motion/trauma), array misrouting, or anatomical challenges (ossification, malformation) [1,2,3,4]. These EEs can lead to stimulation frequencies being assigned to areas outside the cochlea, less effective stimulation in apical regions, or unintended co-stimulation of intracochlear electrodes due to current spread, all of which may compromise clinical outcomes for cochlear implant recipients [1,5,6]. Prevalence estimates of EEs vary by electrode type (lateral-wall electrode arrays versus pre-curved), insertion technique (cochleostomy or round window insertion), timing of assessment (intraoperatively, weeks after surgery, or months later), detection method (e.g., CT scan, X-ray, Impedance measurement) and criteria (e.g., 1+ or 2+ contacts outside of cochlea) [1,7].

Conventional detection of extracochlear electrodes relies on imaging modalities such as high-resolution CT or X-ray, which remain the gold standard for confirming electrode array position and identifying anomalies [1,5,8,9]. However, the routine use of imaging is limited by cost, availability, radiation exposure, and the need for specialized interpretation, and it is not consistently performed as part of standard clinical care. In addition, image quality may be compromised by metallic artifacts from the implant body and electrode contacts and wiring, which may obscure visualization of cochlear structures and the electrode array location [10]. Moreover, determining the localization of individual contacts can be challenging due to the limited resolution of X-ray imaging, and because electrode contact spacing approaches the spatial-resolution limits of clinical CT [11,12,13]. In clinical practice, clinicians may therefore rely on non-imaging indicators of extracochlear electrodes. While high or open impedances at basal electrodes may raise suspicion of EEs and serve as a useful clinical indicator, many EE cases present with normal impedance values [1,14,15]. Consequently, identification often relies on the clinician’s judgement, including the surgeon’s intraoperative visual inspection, postoperative audiological evaluations based on recipients’ feedback, loudness growth for the basal electrode contacts, and electrophysiological measures such as the electrically evoked compound action potential (eCAP). Nevertheless, these approaches have limited sensitivity for detecting EEs and may fail to identify cases in which clinical signs are subtle or absent [2]. As a result, some recipients with EEs may remain undetected in routine clinical practice.

Emerging techniques aim to identify electrode array malposition, including the presence of EEs, by analyzing voltage/impedance measurements recorded on non-stimulated contacts during stimulation of other electrodes [7,14,16,17]. Depending on the cochlear implant manufacturer or literature, this measurement technique is referred to by various names, including Stimulation-Current-Induced Non-Stimulating Electrode Voltages (SCINSEV), Electric Field Imaging (EFI), Trans-Impedance Matrix (TIM), or Impedance Field Telemetry (IFT). In this paper, we will use the term EFI to refer to this method. The principal advantage of using EFI lies in its independence from neural function. Unlike electrophysiological measures, such as eCAP, EFI reflects the physical current spread within the cochlea rather than the response of the auditory nerve [1,16,18]. As an entirely objective and non-behavioral technique, EFI can be performed regardless of a patient’s neural response status. Furthermore, EFI has the potential to identify EEs even in cases where basal electrode impedances (i.e., recorded from the stimulating electrode) remain within normal limits, representing a significant advantage over reliance on impedance measurements alone.

In the past, de Rijk et al. (2020) employed the EFI analysis technique in fresh-frozen human cadaveric heads and achieved 100% sensitivity and 100% specificity in detecting cases with two or more EEs (≥2 EEs). The study, conducted on six temporal bones, provided compelling evidence that EFI-based measurements can differentiate between intracochlear and extracochlear electrode positions, offering a reliable and non-radiological alternative for verifying electrode array placement. In a subsequent study, de Rijk et al. (2022) demonstrated that EFI-based EE detection could be generalized across multiple cochlear implant manufacturers and electrode designs, while also showing its feasibility for intraoperative clinical application in cases of partial insertion and electrode migration. In 2024, Ayas et al. reported a clinical case series involving three patients in whom intraoperative EFI measurements successfully identified EEs in live human subjects, enabling immediate surgical repositioning of the electrode array during the same procedure and potentially reducing the need for postoperative revision surgery and additional radiological imaging [19]. A recent scoping review of impedance measurements in 550 ears, limited to studies involving Cochlear Ltd. devices, reported EEs in 1.45% of cases [7]. However, as the review primarily focused on the clinical applications of impedance measurements rather than the systematic determination of EE prevalence, this occurrence rate may underestimate the true prevalence of EEs. The authors concluded that impedance measurement is a promising intraoperative tool; however, larger normative datasets and standardized interpretation criteria are needed to support broader clinical adoption [7]. Another study by Saoji et al. (2025) examined Advanced Bionics (Advanced Bionics LLC, Valencia, CA, USA) implant recipients, comparing six cases with EEs to ten controls. The average cross impedance difference was higher in the EE group (0.15 kΩ vs. 0.06 kΩ). The authors concluded that EFI is a useful, non-invasive tool for detecting EEs and monitoring for electrode array migration.

Most of the studies mentioned above are small, retrospective, or cadaveric rather than larger clinical cohorts, and heterogeneity in definitions of EEs, electrode array types (lateral wall vs. pre-curved), imaging and electrophysiologic detection methods (impedance measurement, eCAP, spread of excitation) limit comparison across studies. The aim of this study is to evaluate the performance of an optimized automated algorithm for the detection of EEs, validated across both bench and clinical datasets comprising a relatively large sample size.

## 2. Materials and Methods

All datasets included in this study were acquired using Advanced Bionics cochlear implant systems.

### 2.1. Datasets

(a) Bench: The cochlear implant electrode array was submerged in a saline bath to mimic the conductive environment of the cochlea. Normal conditions were simulated with all electrodes immersed in saline (0 EE). Extracochlear conditions were created by removing one to five basal electrode contacts from the saline. For each configuration, three repeated EFI measurements were acquired (*N* = 18 total recordings). While this model produces clear EE conditions, it may also result in elevated or open-circuit impedances at basal electrode contacts located outside the saline bath. Therefore, to evaluate the algorithm under conditions more representative of clinically challenging cases, where EEs may be present despite normal impedance measurements, additional EE scenarios were created using a resistive load model that contains cross-impedances and subsequently tested with the EE detection algorithm (*N* = 2).

(b) Clinical EFI with imaging: EFI measurements were acquired during routine care (AIM™/Target CI), with CT or plain film X-ray (*N* = 226).

(c) A separate EFI dataset that lacked imaging data was used for EE prevalence screening (*N* = 26,692 EFI recordings, unique cases = 6443). These cases had surgery dates that ranged between October 2023 and January 2026. Target CI version 1.5 was used to obtain EFIs for this dataset.

### 2.2. EFI Acquisition

EFI matrices (16 × 16) were recorded while delivering stimulation using a monopolar coupling mode. Each contact was stimulated sequentially while voltages were measured on all contacts with respect to the ground electrode (either case or ring). Impedances were obtained by dividing measured voltages by stimulus current [16,20]. Acquisition used standard clinical settings (e.g., 32 µA; ~18 µs per phase; biphasic, charge-balanced, cathodic-leading current pulses), aligning with prior EFI workflows [21].

### 2.3. Algorithm Overview

An EE detection algorithm was developed, with parameters derived from previously available clinical data separate from the dataset indicated in Section 2.1. The pre-processing and data analysis steps of the algorithm have been detailed below.

#### 2.3.1. Pre-Processing and Exclusion Rules

EFI data require systematic pre-processing to ensure signal integrity and minimize false detection of electrode anomalies. Pre-processing allows the algorithm to analyze meaningful electrical field measurements while reducing the influence of hardware artifacts, telemetry inconsistencies, and measurement noise, thereby improving the reliability and robustness of anomaly detection. Below, the pre-processing steps have been detailed.

- Check if the value of main impedances is less than the threshold level of 0.5 kΩ (invalid)
- Check if there are any short circuits between electrode contacts
- Remove any column and row associated with the index of a short circuit to ground or short between electrode contacts from the EFI
- The Extracochlear algorithm will not be run if:
- There are >2 electrode contacts with invalid impedance measurements or short-circuit electrode contacts
- If the cross-impedance values in the first row exceed 5 kΩ (normal cross-impedance range: ~0.5–2 kΩ), suggesting that the case ground electrode was not in adequate contact with surrounding tissue during EFI recording. This condition may occur intraoperatively, for example due to the presence of an air pocket that impedes effective electrical current flow and usually resolves postoperatively as tissue contact improves.

#### 2.3.2. EE Detection Algorithm

Two sub-schemes are used to determine the presence and number of extracochlear electrode contacts: (1) Slope and (2) Subthreshold.

##### Slope

This part of the algorithm was inspired by the work of de Rijk SR et al. (2020) and is outlined below:

- Cross-impedance values are summed across rows for each column, and the resulting signal is flipped right to left
- The linear fit between all possible segmentations (with a minimum of three elements and a maximum of ten elements for the length of the segment) is calculated
- Best segmentation is determined based on residual calculation (sum of square differences between the linear fit and the original segment)
- The ratio of the slopes of the two most basal segments is calculated
- If the ratio is >1.2, then an extracochlear condition is detected and the number of extracochlear electrode contacts is reported to be the first element of the last segment up to El. 16.

##### Subthreshold

The subthreshold algorithm uses the actual values of cross impedances to determine whether there are any extracochlear electrode contacts or not. If the cross impedance of a particular electrode contact is below 0.4 kΩ in 5 or more rows of the EFI matrix, then that electrode contact is defined as extracochlear.

The algorithm subsequently reports the minimum number of detected extracochlear electrode contacts (EEs) across the two detection schemes.

### 2.4. Outcomes and Statistics

Primary endpoints were defined as: (1) the ability of the proposed method to discriminate between normal and EE conditions in bench and clinical cases with available imaging and (2) agreement between the actual and detected number of EEs in bench and clinical cases with available imaging. For clinical cases without imaging, the observed EE rate was evaluated relative to previously reported prevalence ranges. A two-proportion z-test with pooled variance was conducted to compare the rate of detected EEs between the pre-curved (HiFocus MidScala) and lateral wall (HiFocus SlimJ) electrode types.

## 3. Results

### 3.1. Bench Data

Table 1 summarizes the combined slope and sub-threshold algorithm results for saline and resistive load model data. The saline model was evaluated using the Advanced Bionics HiRes 90K Advantage cochlear implant with a HiFocus 1J electrode array, whereas the resistive load model used the Advanced Bionics HiRes Ultra cochlear implant with a HiFocus SlimJ electrode array. Across the combined bench datasets (*N* = 20), the algorithm achieved 100% sensitivity and specificity in correctly identifying the presence or absence of EEs. In terms of quantifying the number of extracochlear electrodes, the algorithm accurately estimated the count within a margin of ±1 electrode relative to the ground truth.

### 3.2. Clinical Cases with Imaging

In the imaged cohort, algorithmic classification agreed with radiology for the presence or absence of EEs. Misclassification was not observed in this subset (100% sensitivity and specificity). Out of 226 clinical cases, six were identified as having an EE (2.65%). Of these, 5 involved HiFocus SlimJ straight lateral-wall electrode arrays and 1 involved a HiFocus Midscala pre-curved array. Table 2 summarizes the cases based on electrode array type.

**Figure 1:**
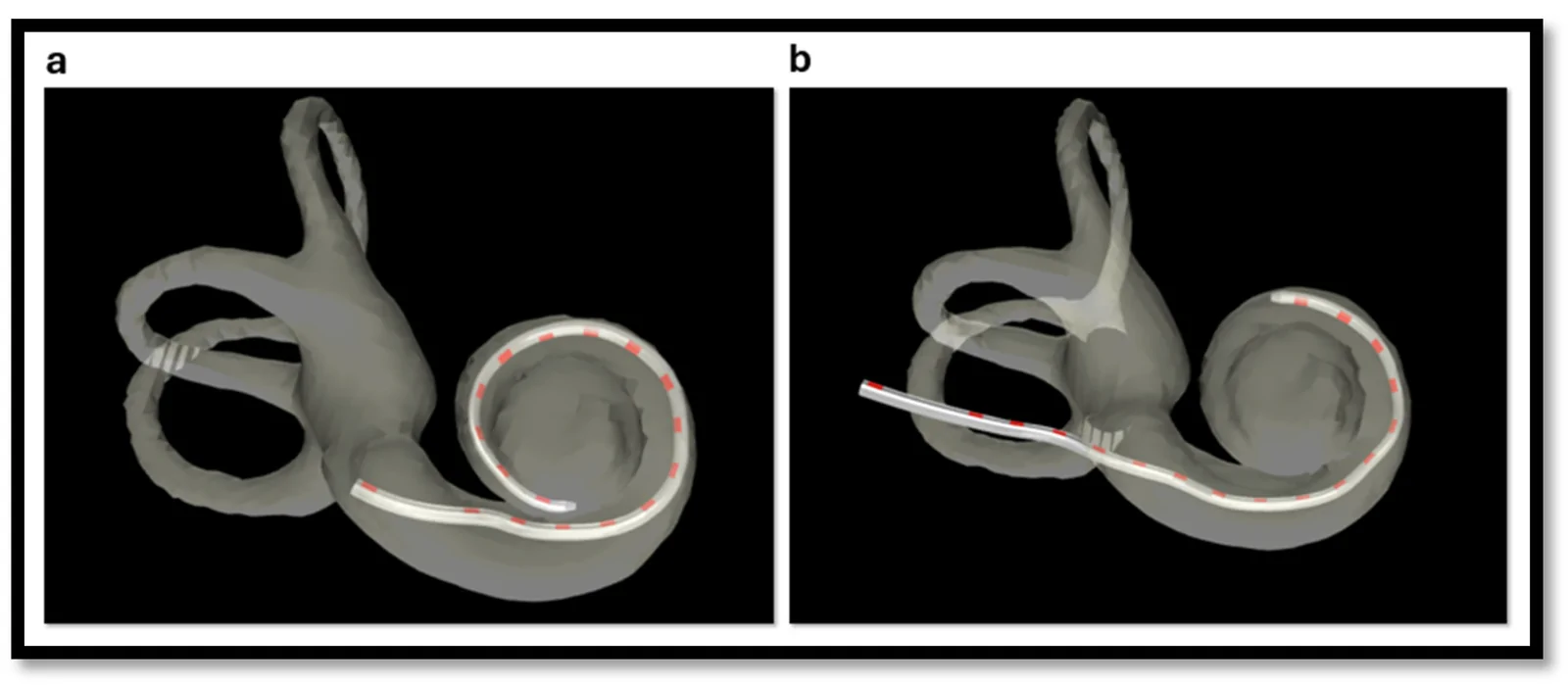
Cochlear implant electrode localization from postoperative CT scans generated using Vanderbilt University’s Oto-pilot system. (**a**) Example of full intracochlear insertion. (**b**) Example of a case with three EEs identified from CT reconstruction.

Figure 1 shows examples of cochlear implant electrode array localization derived from postoperative clinical CT scans. The visualizations were generated using Vanderbilt University’s Oto-pilot system, which automatically localizes cochlear implant electrode arrays in CT images and reconstructs their trajectory within the cochlea with sub-voxel accuracy [2,22,23,24,25]. In Figure 1a, the electrode array demonstrates full intracochlear insertion, whereas Figure 1b illustrates a case with three EEs based on processed CT reconstruction and automated electrode localization by the Oto-pilot system. In comparison, the proposed EE detection algorithm, which only relies on EFI rather than CT imaging, estimated two EEs.

**Figure 2:**
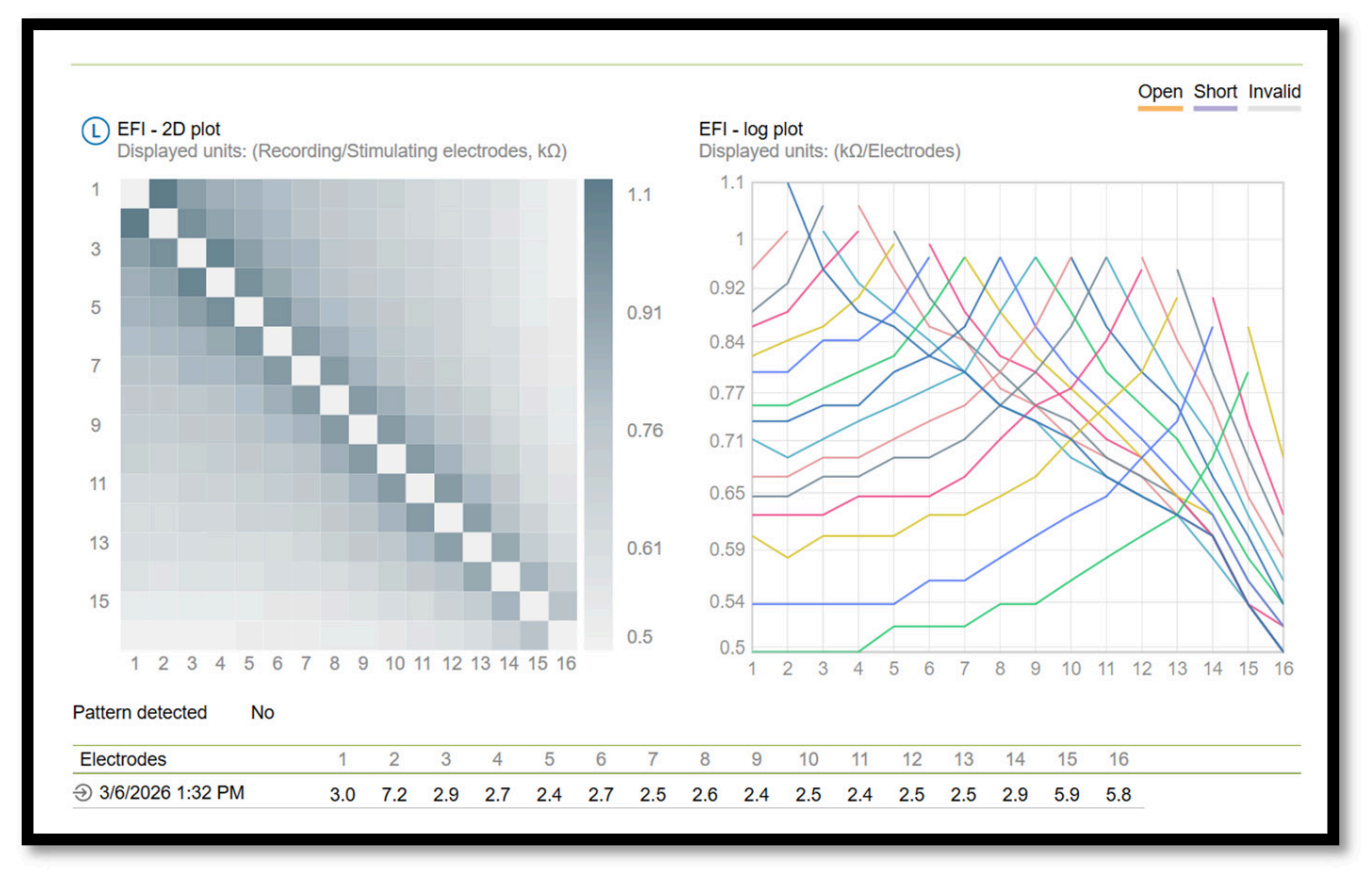
Normal case with full electrode array insertion. The EFI heat map (**left**) shows a gradual decline in cross-impedances with increasing distance from the stimulating electrode contact, consistent with the expected spatial decay of intracochlear current spread. The corresponding EFI line plot (**right**) demonstrates a smooth and continuous distribution of cross-impedance values across electrode contacts, with gradually changing slopes, indicating a consistent conductive environment surrounding all contacts.

**Figure 3:**
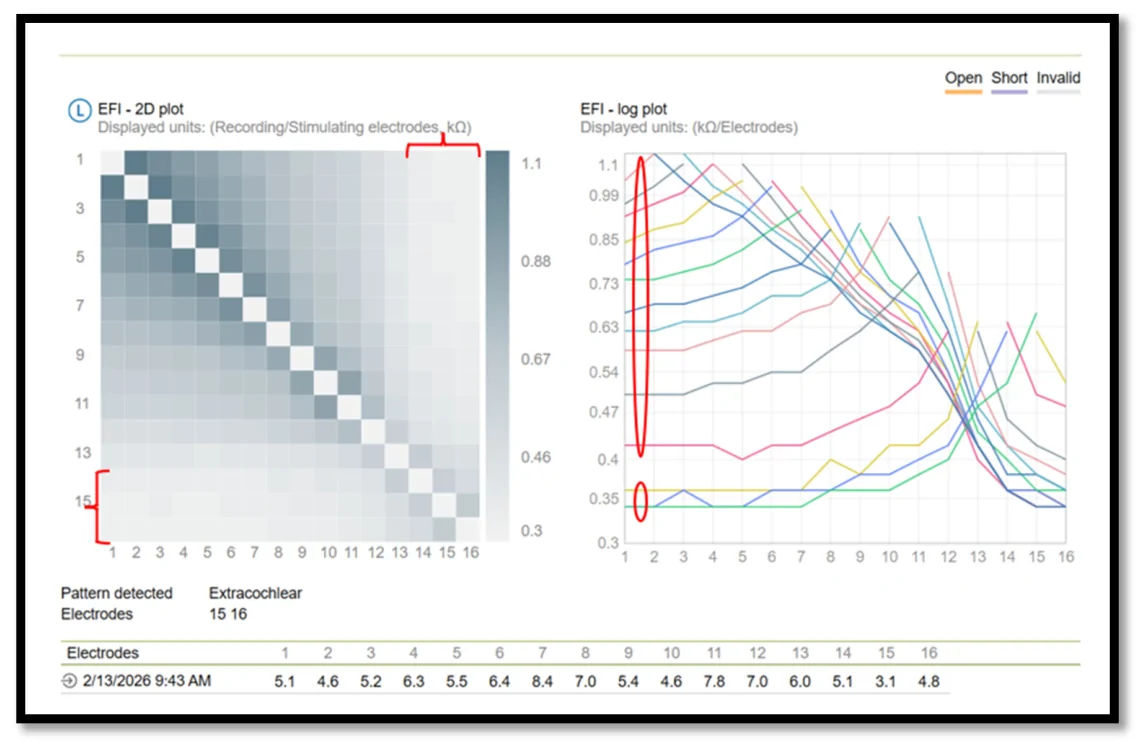
Example of a case containing three EEs. The EFI heat map (**left**) shows regions of lower (light gray) and higher (dark gray) cross-impedance values, indicating disrupted current pathways. The corresponding EFI line plot (**right**) reveals two distinct electrode groups: intracochlear electrodes with higher cross-impedance values, and basal extracochlear contacts with markedly lower cross-impedance values.

The heat maps (2D) and line (Log) plots corresponding to Figure 1a and Figure 1b are shown in Figure 2 and Figure 3, respectively. Examination of the heat map plot for the case with a normal insertion (Figure 2, left panel) reveals a gradual decline in cross-impedance values with increasing distance from the stimulating electrode, reflecting the expected spatial decay of intracochlear current spread. In contrast, the case containing EEs (Figure 3, left panel) exhibits a distinct pattern characterized by regions of lower (light gray) and higher (dark gray) cross-impedance values. The line plots further emphasize this difference. In the normal insertion condition (Figure 2, right panel), the EFI line plots show a smooth and continuous distribution of cross-impedance values across electrodes, with no abrupt transitions between neighboring contacts. In the presence of EEs, the electrode contacts can be visually separated into two groups (Figure 3, right panel). The first group, corresponding to intracochlear electrode contacts, exhibits higher cross-impedance values with a gradual decrease in slope that progressively becomes steeper toward the basal electrodes. In contrast, the second group, corresponding to basal extracochlear contacts, shows markedly lower cross-impedance values and a distinct discontinuity in the EFI profile.

### 3.3. Clinical Cases Without Imaging

The extracochlear electrode detection algorithm was applied to all eligible recordings, defined as those obtained within two months of surgery and without evidence of known implant or electrode array malfunctions such as Ultra V1 electrode interaction or tip fold-over. To ensure that each cochlear implant user was represented only once, recordings were sorted chronologically, and only the most recent recording was retained in cases where multiple measurements were available for the same individual, either within a single session or across different sessions (unique cases). Table 3 summarizes the percentage of EE cases detected by the algorithm, calculated for all eligible EFI recordings (first row) and for unique implant IDs (second row).

Table 4 displays a summary of the unique cases by implant type and electrode-array type. The implant types for these cases were Ultra (*N* = 572), Ultra 3D (*N* = 5870), and HiRes 90K Advantage (*N* = 1). Only one HiRes 90K Advantage implant was identified in the study cohort, reflecting the predominance of newer implant models during the study period (October 2023–January 2026).

As shown in Table 4, the straight lateral-wall (HiFocus SlimJ) array exhibited a higher proportion of detected EE implants (4.77%) compared to the pre-curved (HiFocus Mid-Scala) array (2.54%). To statistically evaluate this difference, a two-proportion z-test was performed. Using the pooled estimate of the population proportion, the test yielded a z-statistic of −4.45 with a corresponding *p*-value of *p* < 0.001, indicating a significant difference between array types. These findings are consistent with previous reports indicating that lateral wall arrays are associated with a significantly higher incidence of extracochlear electrodes than pre-curved arrays [2].

**Figure 4:**
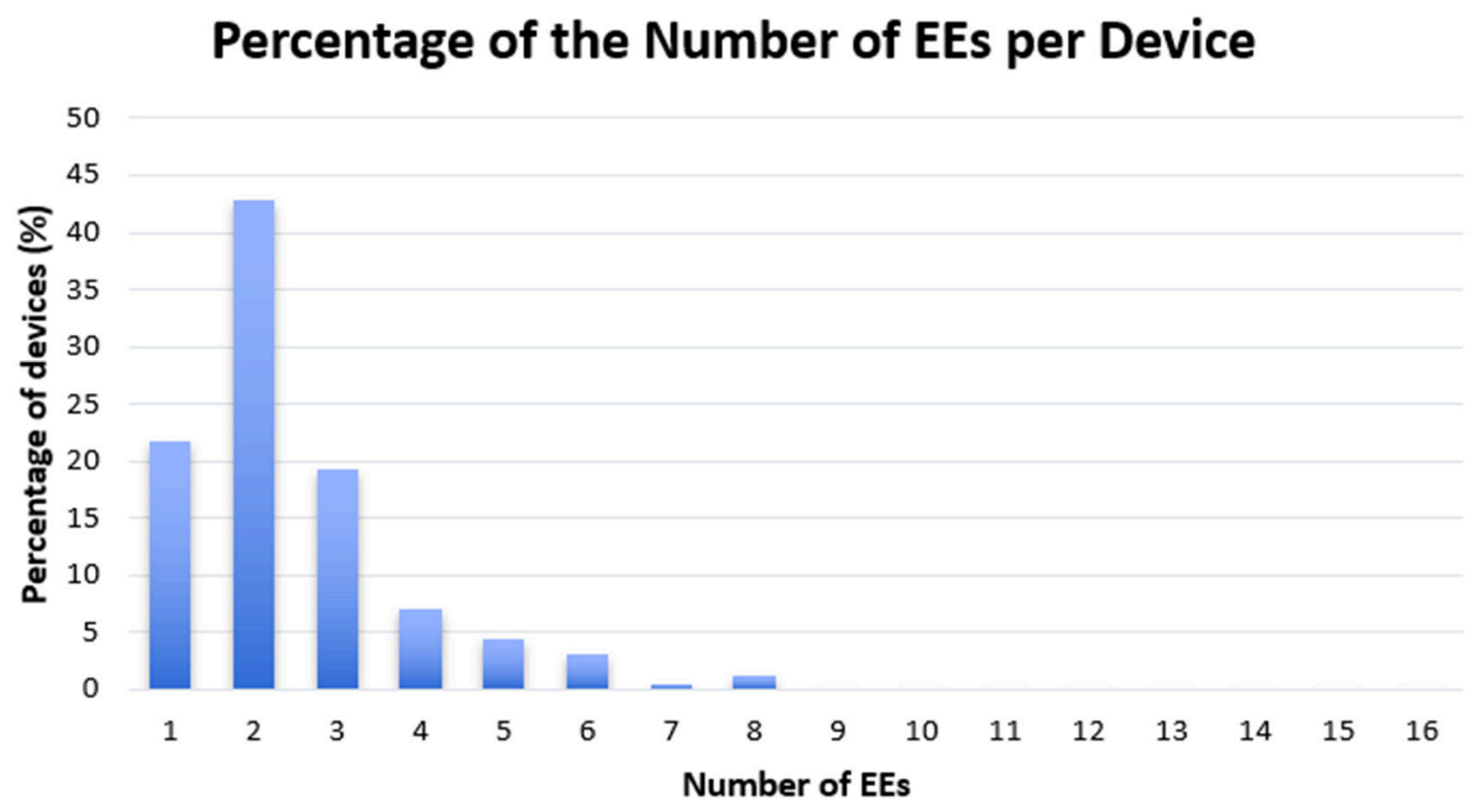
Distribution of the number of EEs per device in the clinical cohort without imaging. Bars represent the percentage of devices with each number of EEs.

Figure 4 illustrates the distribution of the number of EEs per device in the clinical cohort without imaging. As can be seen, the majority of affected devices have one to three EEs, with two extracochlear electrodes accounting for the largest proportion of cases (42.9%). The percentage of devices decreases progressively as the number of EEs increases, indicating that extensive extracochlear placement involving four or more electrodes is relatively uncommon.

## 4. Discussion

This study presents an EFI-based approach for detecting EEs that can be applied both intraoperatively and in the early postoperative period, particularly within the first two months following implantation, when electrode array migration is more likely to occur due to the absence of a stabilizing fibrous sheath around the array [2].

Previous studies have demonstrated the feasibility of using EFI measurements to detect EEs and identify deviations in electrode array positioning. However, the existing literature has largely been limited to small cohorts and cadaveric or retrospective study designs, and previously proposed algorithms were primarily optimized for detecting multiple EEs and failed to successfully identify single EE contact.

Building upon the previous findings, the present study extended prior work in several important ways. An improved analytical approach was developed to increase sensitivity to consider more subtle deviations in current spread, enabling detection of even a single extracochlear electrode contact. This capability is clinically relevant, since the presence of a single EE may indicate early electrode array migration, particularly in the immediate postoperative period, before fibrous encapsulation stabilizes the electrode array. Even a single extracochlear contact can reduce the number of effective stimulation channels, distort tonotopic frequency allocation, and impact mapping. In addition, the dataset was expanded beyond controlled or bench conditions to include a large number of EFI recordings from cochlear implant recipients, with imaging confirmation available for a subset. The inclusion of real-world clinical data is essential, as patient-specific factors, such as anatomical variability, cochlear fibrosis, fluid environment, and electrode array type, can significantly influence EFI signatures.

Relative to prior clinical work, including that of Goh X et al. (2023), who examined electrode array migration using radiographic imaging within the first two weeks post-implantation, the present study incorporates a multicenter dataset and evaluates recordings obtained up to two months postoperatively. This broader temporal window allows for improved detection of both early and delayed migration events, which may otherwise be under-represented in imaging-based studies.

To better interpret the results of the present study, it is important to consider the characteristic EFI patterns associated with intracochlear and extracochlear electrode array positions. Under conditions of full insertion, EFI line plots demonstrate a smooth and continuous distribution of cross-impedance values across the electrode array, without abrupt transitions between neighboring contacts. The gradual change in slope reflects a relatively uniform conductive environment surrounding the electrode contacts, consistent with proper intracochlear positioning. In contrast, recordings containing EEs exhibit a distinctly different pattern, characterized by regions of relatively higher and lower cross-impedance values. This heterogeneous distribution suggests the disruption of normal current pathways, likely due to the transition from the conductive intracochlear environment to surrounding extracochlear media such as air, soft tissue, or fluid. The resulting abrupt change in the EFI profile marks a clear boundary between intracochlear and extracochlear electrode contacts, representing a cross-impedance discontinuity that can be leveraged for detection. This transition is further reflected in the change in slope between the two most basal segments of the summed cross-impedance profile, where the ratio of these slopes indicates reduced interaction between intra- and extracochlear electrode contacts, consistent with decreased cross-impedance values at the basal electrodes.

The prevalence of EEs observed in the present study is consistent with previously reported findings in the literature. Prior work has demonstrated that electrode array migration and extracochlear positioning, while relatively uncommon, are clinically relevant phenomena that may occur at the time of insertion, or across a broad postoperative time window (within weeks after surgery, or months later) and may even remain asymptomatic in some cases, for example, those cases where migration occurred after any post-operative imaging was conducted but before first fitting. Across the literature, the occurrence of EEs shows substantial variability, with reported rates spanning a wide range depending on the cohort and detection approach. Imaging-based assessments tend to report higher occurrence when electrodes are systematically evaluated postoperatively, whereas lower rates are typically reported when detection relies on clinical symptoms or routine programming measures [2]. In addition, several studies indicate that a proportion of these cases remain clinically silent and may go unrecognized without objective assessment, suggesting that the true occurrence may be underestimated in standard clinical practice [8,26]. Pooled estimates indicate an overall occurrence of approximately 6–7%, reflecting migration events identified across a broad postoperative time course, from the early postoperative weeks to several years after implantation [8]. In this context, the EE occurrence rate found in our study is in line with reported literature estimates for EE prevalence, supporting the external validity of the present findings [8,17].

With respect to electrode array design, a statistically significant difference between designs was noted in cases without imaging. This finding aligns with prior literature indicating that electrode array design influences insertion dynamics and the likelihood of malposition. In particular, lateral-wall electrode arrays appear more susceptible to extracochlear positioning or postoperative electrode contact migration compared to pre-curved arrays, which may better conform to cochlear anatomy [2,26,27,28].

The integration of an extracochlear electrode contact detection algorithm into clinical fitting software could enable clinicians to benefit from EFI-based screening, even when imaging is not part of routine clinical care. Intraoperatively, EFI monitoring can alert the surgeon to incomplete insertion or early electrode array migration, allowing correction before closure, or prompting perioperative imaging when an anomaly is detected. In the postoperative period, identification of EEs is critical because they can lead to inappropriate frequency-to-electrode assignments, leading to a loss of important high-frequency information. Routine EFI monitoring can detect positional changes over time and guide appropriate interventions, including deactivation of basal contacts, re-imaging, remapping, or surgical revision when necessary.

## 5. Limitations

A key limitation of this study is the limited size of the radiologically validated cohort (226 cases, including 6 imaging-confirmed EE cases). Accordingly, the reported performance metrics should be interpreted with caution and validated in larger radiologically confirmed cohorts. In addition, the clinical dataset without imaging lacked imaging confirmation, as well as relevant clinical metadata, including the stage at which EFI measurements were collected (intraoperative versus postoperative), implantation status (primary implantation versus re-implantation), patient etiology, and electrode programming history (e.g., whether suspected EEs had been deactivated in clinical maps). These limitations restrict definitive validation and comprehensive clinical characterization of the detected EE contacts in the dataset of clinical data without imaging. In addition, long-term clinical outcomes, such as speech perception performance, were not available and were not within the primary scope of this study; therefore, their relationship with the presence of EEs could not be evaluated.

It is noteworthy to mention that, although advanced impedance screening integrated into clinical fitting software identifies open, short, and invalid electrode contacts, careful consideration is required, as these conditions may contribute to false-positive detections of EEs. Accordingly, the proposed algorithm should not be considered a definitive diagnostic tool, but rather as a supportive screening method to guide clinical decision-making, such as determining the need for further psychophysical or imaging evaluation.

## 6. Conclusions

An EFI-based algorithm has been demonstrated to reliably and rapidly screen for extracochlear electrode contacts using routine clinical measurements. Bench, resistive load model, and initial clinical validations support its promise as an intra-operative and early postoperative screening tool that complements imaging and may help optimize CI recipients’ outcomes.

## Figures and Tables

**Table 1:** Comparison of actual EEs and algorithm-detected EEs in bench measurements.

Bench Data	Actual EE Number	Algorithm Result
Resistive Load Model	13,14,15,16	13,14,15,16
Resistive Load Model	13,14,15,16	13,14,15,16
Saline	16	16
Saline	16	15,16
Saline	16	16
Saline	15,16	14,15,16
Saline	15,16	15,16
Saline	15,16	15,16
Saline	14,15,16	14,15,16
Saline	14,15,16	13,14,15,16
Saline	14,15,16	14,15,16
Saline	13,14,15,16	13,14,15,16
Saline	13,14,15,16	13,14,15,16
Saline	13,14,15,16	13,14,15,16
Saline	12,13,14,15,16	12,13,14,15,16
Saline	12,13,14,15,16	12,13,14,15,16
Saline	12,13,14,15,16	12,13,14,15,16
Saline	No EE	No EE
Saline	No EE	No EE
Saline	No EE	No EE

**Table 2:** Percentage of Detected EEs in Clinical Cases with Imaging.

Electrode Type	Implant Count	Number of Detected EEs	Percentage
HiFocus Midscala	115	1	0.87%
HiFocus SlimJ	111	5	4.5%
Total	226	6	2.65%

**Table 3:** Prevalence of detected EEs across all eligible recordings and unique implant IDs.

Sample	Recordings Count	Number of EEs Detected	Percentage
All eligible recordings	26,692	1373	5.14%
Unique implant ID	6443	254	3.94%

**Table 4:** Prevalence of extracochlear electrode (EE) detections by implant and electrode type across unique implant IDs.

Sample	Implant Type	Electrode Type	Implant Count	Implants with EE Cases	Percentage (%)
Unique implant ID	HiRes 90K Advantage = 1	HiFocus Midscala	2397	61	2.54
	Ultra = 43				
	Ultra 3D = 2353				
	Ultra = 529	HiFocus SlimJ	4046	193	4.77
	Ultra 3D = 3517				

## Data Availability

Aggregated data supporting the findings is not accessible due to privacy and legal constraints.
